# Ab Initio Insight into the Interaction of Metal-Decorated Fluorinated Carbon Fullerenes with Anti-COVID Drugs

**DOI:** 10.3390/ijms23042345

**Published:** 2022-02-21

**Authors:** Konstantin P. Katin, Alexey I. Kochaev, Savas Kaya, Fadoua El-Hajjaji, Mikhail M. Maslov

**Affiliations:** 1 Laboratory of Computational Design of Nanostructures, Nanodevices, and Nanotechnologies, Research Institute for the Development of Scientific and Educational Potential of Youth, Aviatorov Str. 14/55, 119620 Moscow, Russia; a.kochaev@ulsu.ru (A.I.K.); MMMaslov@mephi.ru (M.M.M.); 2 Institute of Nanotechnologies in Electronics, Spintronics and Photonics, National Research Nuclear University “MEPhI”, Kashirskoe Shosse 31, 115409 Moscow, Russia; 3 Research and Education Center “Silicon and Carbon Nanotechnologies”, Ulyanovsk State University, 42 Leo Tolstoy Str., 432017 Ulyanovsk, Russia; 4 Department of Chemistry, Faculty of Science, Cumhuriyet University, Sivas 58140, Turkey; savaskaya@cumhuriyet.edu.tr; 5 Engineering Laboratory of Organometallic, Molecular Materials, and Environment, Faculty of Sciences, University Sidi Mohamed Ben Abdellah, Fez 1796, Morocco; fadoua.elhajjaji@usmba.ac.ma

**Keywords:** fullerenes, drug delivery, COVID-19, favipiravir, chloroquine, density functional theory

## Abstract

We theoretically investigated the adsorption of two common anti-COVID drugs, favipiravir and chloroquine, on fluorinated C_60_ fullerene, decorated with metal ions Cr^3+^, Fe^2+^, Fe^3+^, Ni^2+^. We focused on the effect of fluoridation on the interaction of fullerene with metal ions and drugs in an aqueous solution. We considered three model systems, C_60_, C_60_F_2_ and C_60_F_48_, and represented pristine, low-fluorinated and high-fluorinated fullerenes, respectively. Adsorption energies, deformation of fullerene and drug molecules, frontier molecular orbitals and vibrational spectra were investigated in detail. We found that different drugs and different ions interacted differently with fluorinated fullerenes. Cr^3+^ and Fe^2+^ ions lead to the defluorination of low-fluorinated fullerenes. Favipiravir also leads to their defluorination with the formation of HF molecules. Therefore, fluorinated fullerenes are not suitable for the delivery of favipiravir and similar drugs molecules. In contrast, we found that fluorine enhances the adsorption of Ni^2+^ and Fe^3+^ ions on fullerene and their activity to chloroquine. Ni^2+^-decorated fluorinated fullerenes were found to be stable and suitable carriers for the loading of chloroquine. Clear shifts of infrared, ultraviolet and visible spectra can provide control over the loading of chloroquine on Ni^2+^-doped fluorinated fullerenes.

## 1. Introduction

Carbon-based nanomaterials interact with a variety of pharmaceutical drugs [1]. Fullerenes are the smallest carbon nanostructures. They are hollow spherical cages, and *sp*^2^-hybridized carbon atoms form pentagons and hexagons on their surface [2,3]. Fullerenes are quite stable and soluble in organic solvents. The functionalization of fullerenes by various radicals provides a wide variation in their solubility. Fullerenes consist of an even number of atoms. The smallest fullerene, C_20_, has a strained skeleton consisting of 12 pentagons [4]. However, it has a high kinetic stability [5]. Giant fullerenes can contain several hundred of atoms [6,7]. However, fullerenes C_60_ and C_70_ (especially C_60_) are the most stable and are obtained with the highest yield [7].

Carbon fullerenes are considered promising nanoparticles for drug delivery [8]. The most common fullerene, C_60_, has a well-reproducible stable carbon skeleton. In contrast to other *sp*^2^-hybridized carbon nanostructures, such as nanotubes and graphene flakes, the shape and size of C_60_ can be easily reproduced from the experimental point of view. The chirality of nanotubes and the exact size of nanoflakes are difficult to control, whereas C_60_ fullerene has a well-defined structure. In addition, the curvature of the fullerene surface provides its increased chemical activity [9,10,11]. Like other carbon nanostructures, fullerenes are not toxic to the human body [12,13,14]. The doping of fullerenes results in a wide family of derivative compounds, which possess a wide variety of physical and chemical characteristics, including adsorption properties, electronic structure and solubility [15,16,17].

There are three common ways of fullerenes doping. Endohedral doping involves the formation of guest-host complexes, in which the doping atom or molecule cannot leave the fullerene cage due to spatial difficulties. Substitution doping involves the embedding of foreign atoms into the fullerene skeleton. This type of doping is very common for drug delivery applications since the embedded atom becomes the reaction centre to which the drugs can attach. In particular, fullerenes doped with various elements, including silicon [18], germanium, boron and nitrogen [19], metals [20,21] and other elements [22,23,24], were considered for drug delivery. External doping implies the formation of the chemical bond between the carbon atom and the doping functional group. A significant advantage of such kind of doping is that it does not require the destruction of the carbon skeleton of fullerene. Despite the strained structure of fullerene, the breaking of carbon–carbon bonds in its skeleton requires high activation energy of about 7 eV [25] and, therefore, high temperatures or very active reagents. Thus, external doping seems to be the “gentlest” type of doping, providing good reproducibility of the structure of doped fullerenes.

Fluorine is a very suitable dopant for carbon nanoparticles used as the drug carriers. This very electronegative element forms strong hydrogen bonds with drug molecules. Recently, synthesized and approbated fluorinated graphene nanoflakes have demonstrated other advantages [26,27,28]. The carbon–fluorine bonds provide greater activity in the near-infrared range, providing heat delivery to the system (the heat is necessary for the release of drugs and additional therapeutic effects [29]). In addition, fluorine helps to track nanoparticles inside the human body using nuclear magnetic resonance [30]. For these reasons, fluorinated fullerenes appear to be the preferred carriers of drugs compared to pristine ones.

Fluorinated fullerenes do not have free chemical bonds; therefore, they can interact with drugs molecules only through hydrogen or van-der-Waals bonds. Stronger bonds are possible via the introduction of metal ions. These ions act as linkers, forming complexes with drug molecules and fullerenes. In Ref. [31], the interaction of metal-decorated fullerenes with several drugs that presumably exhibit anti-COVID19 activity was investigated. The authors considered five promising transition metals—Ti, Cr, Fe, Ni, and Zn. Among them, three metals (Cr, Fe, Ni) demonstrated the best interaction with drug molecules [31]. Note that the presence of negatively charged fluorine atoms in the system can contribute to the attraction between metal ions and fullerenes.

**Figure 1:**
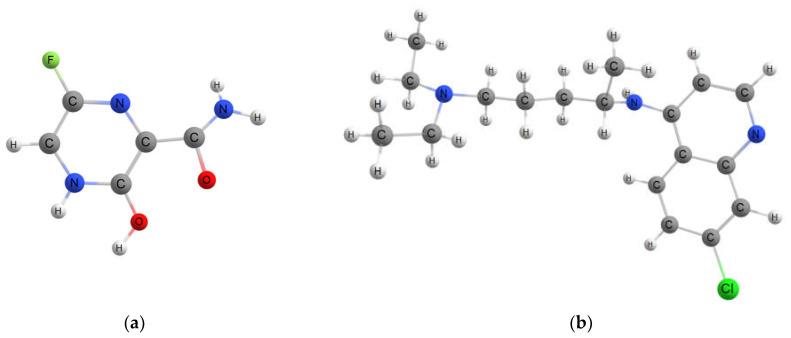
Chemical structures of (**a**) favipiravir and (**b**) chloroquine molecules.

In this paper, we examined the effect of fluorination on the interaction of fullerenes with metal ions and two anti-COVID19 drugs, favipiravir and chloroquine. Their structures are presented in Figure 1. According to recent studies, “favipiravir has shown rapid viral clearance and faster clinical improvement” [32]. It was approved in many countries, including Russia and India, for COVID-19 treatment. Recent studies also suggest the high therapeutic efficiency of chloroquine [33,34]. Note that the adsorption of both drugs on pristine and doped fullerenes was intensively investigated [19,21,35,36], but the effect of fullerene fluorination was not considered so far. Here we applied the electron density functional theory to clarify the structural and spectroscopic features of fluorinated fullerenes decorated with previously selected metal ions (Cr, Fe, Ni) and investigate their interaction with two mentioned drugs. The rest of the article is organized as follows. Section 2 describes the results of the study. Section 3 is devoted to the description of computational details and methods. Section 4 contains the conclusion.

## 2. Results and Discussion

### 2.1. Atomistic Models of Fluorinated Fullerenes

The concentration of fluorine strongly affects the interaction of fluorinated fullerene with ions and drugs. At a low fluorine concentration, the fullerene surface remains partially free and can participate in the interaction with drugs. On the other hand, all fullerene surfaces are coated with fluorine radicals at a higher fluorine concentration. To cover both cases, we considered two fluorinated isomers, C_60_F_2_ and C_60_F_48_. They correspond to the lowest and the highest fluorinated fullerenes, respectively, experimentally observed in significant yields [37]. The structures of these models are described in computational details section.

### 2.2. Interaction of Drugs Molecules with the Pristine and Fluorinated Fullerenes

According to previous studies, the drugs interact poorly with the pristine C_60_. The binding energy between C_60_ and favipiravir in the gas phase is as low as 0.3 eV, and the presence of solvents changes it only by about 10% [35]. Chloroquine does not contain active oxygen atoms and, therefore, interacts even weaker with C_60_, possessing the binding energy of about 0.06 eV [21]. Therefore, any modification of pristine C_60_ is necessary for efficient drugs loading.

**Figure 2:**
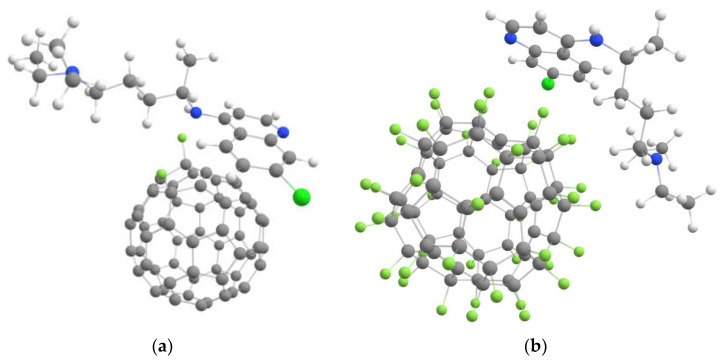
Optimized geometry of the chloroquine molecule loaded on fluorinated fullerenes (**a**) C_60_F_2_ and (**b**) C_60_F_48_.

First, we investigated the interaction of fluorinated fullerenes C_60_F_2_ and C_60_F_48_ with favipiravir. During geometry optimizing, hydroxyl groups of favipiravir become near fluorine atoms to form O-H..F hydrogen bonds. However, we observed the detachment of fluorine from fullerene and the forming of the HF acid after that. We tried many different initial positions of fullerene and the drug, but we always observed the formation of HF as a result of optimization. Similar defluorination of fullerene by OH groups was observed experimentally [38]. Thus, favipiravir results in defluorination of fullerene. Therefore, fluorinated fullerenes are not a suitable drug carrier for favipiravir and similar drugs with active OH groups. In contrast, we observed the successful loading of chloroquine on both C_60_F_2_ and C_60_F_48_ (see Figure 2).The calculated results are collected in Table 1. One can see that the presence of fluorine leads to an increase in the binding energy by more than ten times. For low-fluorinated fullerene C_60_F_2_, electrostatic attraction between nitrogen and fluorine combines with π-π interaction of non-coated fullerene surface with aromatic rings of chloroquine (the distance between parallel aromatic rings belong to fullerene and drug is about 3.3 Å). As a result, low-fluorinated fullerene C_60_F_2_ possesses almost as strong binding to the drug as high-fluorinated fullerene C_60_F_48_. Note that slight interaction of fluorine with carbon aromatic rings was reported in Ref. [39]. The stronger distortion of the chloroquine in the C_60_F_48_ + drug complex can be explained by the attraction of the non-aromatic part of the drug to fluorine. However, distortions of both fullerenes and drugs are quite slight, and their total contribution to interaction energy does not exceed 10%. Electron localization function for both complexes presented in Figure 2 are plotted in Supplementary materials (Figure S1a,b).

### 2.3. Interaction of Metals Ions Molecules with the Pristine and Fluorinated Fullerenes

**Figure 3:**
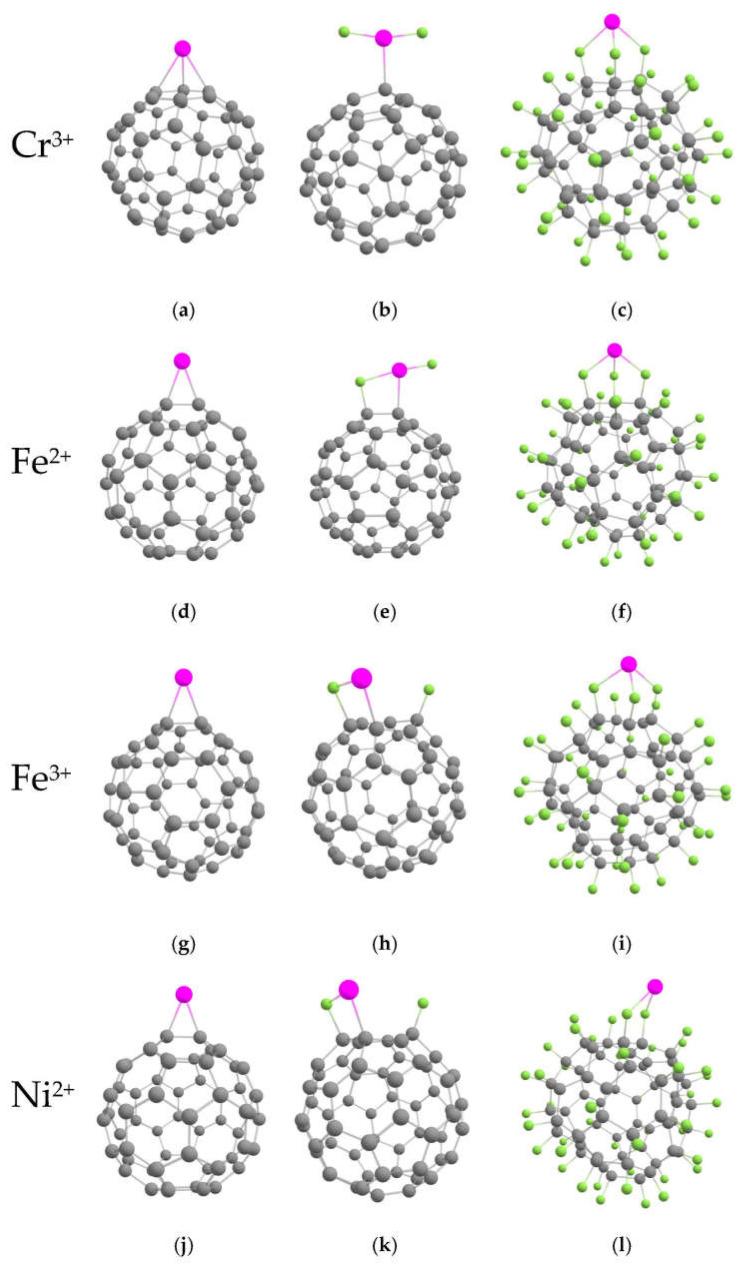
Optimized geometries of pristine, low- and high-fluorinated C_60_ decorated with metal ions: (**a**) C_60_Cr^3+^, (**b**) C_60_F_2_Cr^3+^, (**c**) C_60_F_48_Cr^3+^, (**d**) C_60_Fe^2+^, (**e**) C_60_F_2_Fe^2+^, (**f**) C_60_F_48_Fe^2+^, (**g**) C_60_Fe^3+^, (**h**) C_60_F_2_Fe^3+^, (**i**) C_60_F_48_Fe^3+^, (**j**) C_60_Ni^2+^, (**k**) C_60_F_2_Ni^2+^, (**l**) C_60_F_48_Ni^2+^.

Binding energies between chloroquine drug and fluorinated fullerenes considered above are lower than 0.5 eV. Such energies are insufficient to provide durable drug loading. Therefore, the introduction of metal ions is required. Cr, Fe, and Ni were recognized as the best metals for doping fullerene-based drug carriers [31]. Therefore, we considered the interaction of Cr^3+^, Fe^2+^, Fe^2+^, and Ni^2+^ ions with pristine and fluorinated C_60_. We tried different configurations of doping ions (top, bridge, hollow) and selected ones with the lowest energies. Resulted geometries are shown in Figure 3, whereas corresponding characteristics are presented in Table 2.

We found that all ions strongly interacted with the carbon cage. The ion formed three bonds with carbon atoms, whereas others ions preferred a bridge configuration under the centre of the C-C bond. Note that the observed behaviour of charged ions in solution differs from the behaviour of neutral atoms in a vacuum (for example, neutral Ni atoms prefer a hollow position on fullerene [40]). One can see that ions with a charge of +3 possess much higher Eb values than ions with the charge of +2. It is a remarkable fact that the low concentration of fluorine results in the increase of the *E*_b_ values for all metal ions. Preferable positions of metal ions on low-fluorinated fullerenes are located near the fluorine atoms. However, Cr^3+^ and Fe^2+^ ions lead to the detachment of fluorine from the carbon cage (see Figure 3). In highly fluorinated fullerene, the carbon surface is completely coated. Therefore, metal ions bind only to fluorine. The nickel ion forms two bonds with fluorine atoms, whereas all other ions form three bonds each. The binding energy, in this case, is significantly lower than for low-fluorinated fullerenes (see Table 2). 

Deformation energies of fullerenes C_60_F_n_ (*n* = 0, 2, 48) were calculated as energy differences between distorted and relaxed fluorinated fullerenes, as was described above. We were not surprised that the deformation energies are significantly higher for fluorinated fullerenes, in which fluorine atoms can rotate to adjust to doping ions. 

It should be noted that the nickel ion Ni^2+^ seems to be the most suitable for doping fullerene. This ion does not threaten the stability of fluorinated fullerene and demonstrates moderate binding energy, which is sufficient for reliable adsorption on fullerene.

### 2.4. Loading of Chloroquine on Metal-Decorated Fluorinated Fullerenes

We examined the interaction of the chloroquine drug with the pristine and fluorinated fullerenes doped with metal ions. We observed the formation of the covalent M–N bond between the metal ion M and the drug. Corresponding bonds lengths and other calculated characteristics of fluorinated fullerenes doped with metal ions and loaded with chloroquine are presented in Table 3. We were not able to consider complexes containing C_60_F_2_Cr^3+^ and C_60_F_2_Fe^2+^ compounds, because Cr^3+^ and Fe^2+^ ions induce fullerene defluorination, as described in above. 

**Figure 4:**
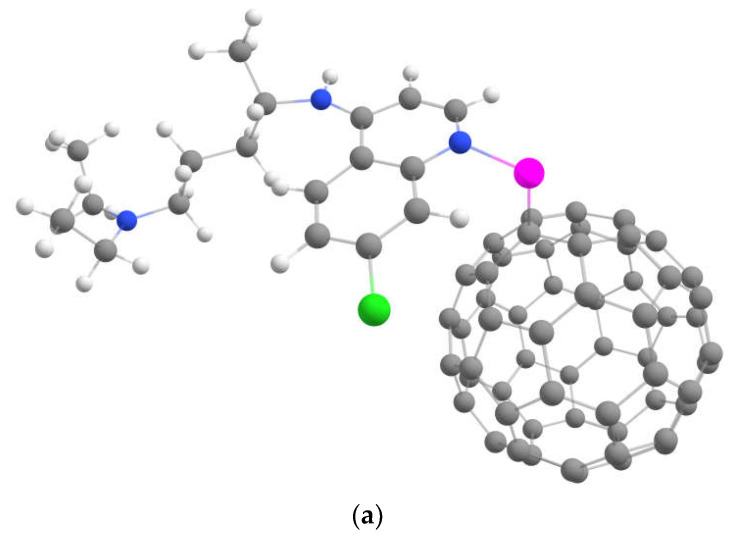

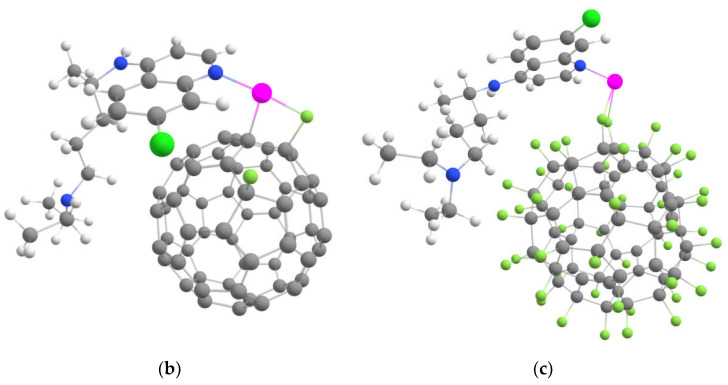
Optimized geometries of chloroquine drug loaded on Ni^2+^-decorated fluorinated fullerenes (**a**) C_60_Ni^2+^, (**b**) C_60_F_2_Ni^2+^ and (**c**) C_60_F_48_Ni^2+^.

Note that a high concentration of fluorine significantly enhances the binding between the metal-doped fullerenes and the drug. Complexes containing Ni^2+^ ion demonstrated a moderate binding with the drug, suitable for reliable loading. The geometries of these complexes are shown in Figure 4. This figure confirms that both covalent and non-covalent interactions occur between the drug and fullerene.

Deformation energies of the drug and curriers are also presented in Table 3. One can see that they make up a significant proportion of the binding energy. Deformation energies of high-fluorinated fullerenes are substantially higher. This fact indicates that high-fluorinated fullerenes can “adjust” their shapes to the drug. In most cases, fullerenes doped with Ni^2+^ ion provide the lowest deformation of both drug and carrier, as indicated in Table 3.

### 2.5. Spectral Fingerprints of the C_60_F_2_Ni^2+^-Chloroquine and C_60_F_48_Ni^2+^-Chloroquine Complexes

**Figure 5:**
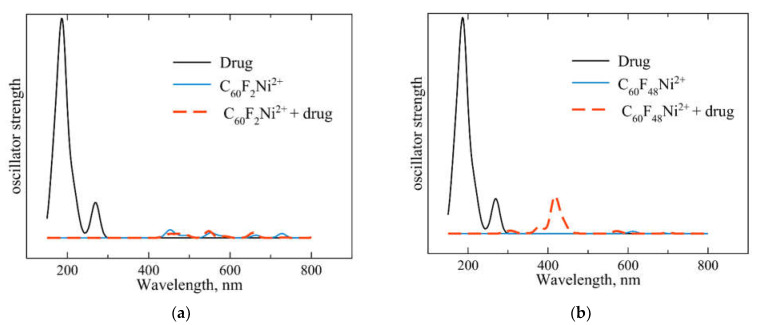
Ultraviolet and visible spectra of (**a**) chloroquine drug (black line), C_60_F_2_Ni^2+^ carrier (blue line) and carrier + loaded drug complex (red dashed line). (**b**) The same spectra for high-fluorinated C_60_F_48_Ni^2+^ carrier loaded with chloroquine. The calculated wavelengths corresponding to the transitions are broadened by Gaussian curves with *σ* = 10 nm.

Here we considered spectral features of Ni-doped fluorinated fullerenes loaded with chloroquine. We chose nickel as the best doping element, providing moderate binding energies and low drug deformations and not leading to carrier defluorination. The formation of a new M-N bond and the deformation of molecules associated with the drug loading should affect the optical spectra of the considered systems. Thus, one can use spectroscopic methods to control the process of loading. Figure 5 shows the ultraviolet and visible spectra of the drug, carriers and corresponding complexes. It can be seen that the activity of the drug in the high-energy region (200–300 nm) disappears when it loads on fullerene. In addition, the formation of the “C_60_F_48_Ni^2+^ + chloroquine” complex is accompanied by the appearance of a peak at 418 nm.

**Figure 6:**
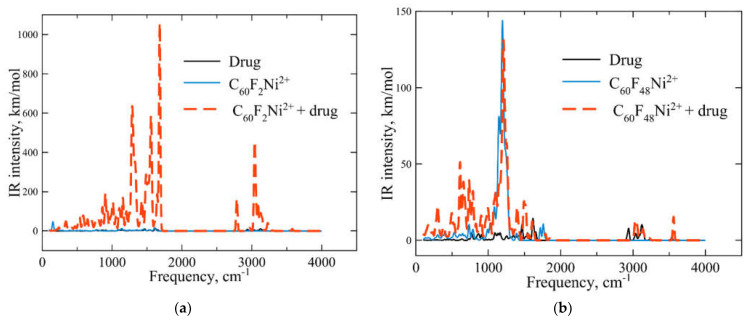
Infrared spectra of (**a**) chloroquine drug (black line), C_60_F_2_Ni^2+^ carrier (blue line) and carrier + loaded drug complex (red dashed line). (**b**) The same spectra for high-fluorinated C_60_F_48_Ni^2+^ carrier loaded with chloroquine. The calculated wavelengths corresponding to the transitions are broadened by Gaussian curves with *σ* = 10 cm^−1^.

Figure 6 shows the active infrared frequencies and the corresponding intensities for the systems under consideration. It can be seen from Figure 6 that the spectrum of the “carrier + drug” system is not a simple sum of the spectra of its parts. Therefore, one can control the loading process by spectral measurements. Integral infrared absorption intensity of the “C_60_F_2_Ni^2+^ + drug” complex is much higher than absorption intensities of its parts (see Figure 6a). Therefore, drug loading on C_60_F_2_Ni^2+^ can be easily indicated. Loading the drug onto the C_60_F_48_Ni^2+^ cluster does not lead to such a significant change in the infrared spectrum. However, drug loading on C_60_F_48_Ni^2+^ results to an appearance of a series of active frequencies in the range of 590–700 cm^−1^.

## 3. Computational Details

For C_60_F_2_, we constructed the most stable isomer, in which two fluorine atoms occupy the same carbon hexagon in the *para* position [41]. For C_60_F_48_, we selected the low-energy isomer with D3 symmetry, which provides the best fit of the X-ray diffraction spectra of C_60_F_48_ [42]. For geometry optimizing, we used B3LYP exchange–correlation functional, which is widely used for investigations of drugs on fullerenes (see, for example, [43,44]). Two Pople’s electronic basis sets 6–31 G*_ldz [45] and 6–31 G** [46], were consistently used for metal ions and other atoms, respectively. Grimme’s D3 corrections [47] were introduced to take into account non-covalent dispersion interactions. The solvent effect was simulated with the conductor-like polarization model COSMO [48]. We assumed that the solvent is water with a dielectric constant of 78.4. Note that all calculations, including geometry optimizing, were carried out by taking the solvent into account. To accelerate the calculations rate, we combined the power of GPU-based TeraChem software [49] with the efficiency of the geomeTRIC optimizer [50]. Vibrational frequencies and infrared (IR) spectra were calculated with the same B3LYP functional. Ultraviolet and visible spectra (UV–Vis) were calculated with CAM-B3LYP functional, which is more suitable for such calculation due to taking into account the long-range Coulomb correlations [51]. Twenty excited states were considered with the Tamm-Dancoff time-dependent density functional approach [52].

The binging energies were calculated as follows. To characterize chloroquine–fullerene interaction quantitatively (Section 2.2, Table 1), we calculated binding energies *E*_b_ as:

*E*_b_ = *E*(C_60_F_n_) + *E*(chloroquine) − *E*(C_60_F_n_ + chloroquine) + BSSE. (1)

Here *n* = 2 or 48 for low- and high-fluorinated fullerene, respectively. We also calculated deformation energies *E*_def_ as the energy differences between distorted fullerene/drugs included into the complex and relaxed fullerene/drugs. The total interaction energy between distorted fullerene and drugs can be calculated as a sum of three components:

*E*_int_ = *E*_b_ + *E*_def_(C_60_F_n_) + *E*_def_(chloroquine). (2)

To characterize the interaction of pristine or fluorinated fullerene with a metal ion (Section 2.3, Table 2), binding energies *E*_b_ were calculated as

*E*_b_ = *E*(C_60_F_n_) + *E*(M ion) − *E*(C_60_F_n_M ion) + BSSE. (3)

Here *n* = 0, 2 or 48 for pristine, low- and high-fluorinated fullerene, respectively. For metal-decorated fluorinated fullerenes loaded with drugs (Section 2.4, Table 3), the binding energies *E*_b_ were calculated as

*E*_b_ = *E*(C_60_F_n_M ion) + *E*(drug) − *E*(complex) + BSSE. (4)

Here *n* = 0, 2 or 48 for pristine, low- and high-fluorinated fullerene, respectively; M = Cr^3+^, Fe^2+^, Fe^3+^, Ni^2+^. In Formulas (1), (3), and (4), basis set superposition error (BSSE) were taken into account with the ghost atoms method implemented in TeraChem.

## 4. Conclusions

Carbon fullerene derivatives are interesting because they combine the advantages of the pristine fullerene (controlled size, high stability and biocompatibility) with additional improvements related to dopants. Systems with two different types of dopants are particularly complex since it is necessary to consider the interaction of dopants with each other. Here we have considered fullerenes functionalized simultaneously by fluorine and metal ions. It is not surprising that not all of the considered systems turned out to be stable: Cr^3+^ and Fe^2+^ ions led to the rupture of C–F bonds. In contrast, Fe^3+^ and Ni^2+^ ions are compatible with fluorinated fullerenes. However, Fe^3+^ can be reduced to Fe^2+^ under the action of antioxidants contained in the human body [53]. Therefore, we selected the only suitable ion, Ni^2+^, which does not violate the structures and stability of fluorinated fullerenes. Therefore, Ni-doped fluorinated fullerenes combine high adsorption to drugs due to the presence of the Ni^2+^ ion with well-known advantages of fluorinated carbon nanosystems. We expect that such complexes can be considered a basis for carriers suitable for chloroquine and other drugs with similar molecular structures.

## Figures and Tables

**Table 1:** Calculated geometric and energetic characteristics of fluorinated C_60_ loaded by chloroquine drug. Binding energy *E*_b_ (eV), fullerene and drug deformation energies *E*_def_ (eV), interaction energy *E*_int_ (eV), dipole moments *D* (Debye), frontier molecular orbitals HOMO and LUMO (eV) and HOMO–LUMO gaps (eV) are presented.

	*E*_b_	*E*_def_ (Fullerene)	*E*_def_ (Drug)	*E*_int_	*D*	HOMO	LUMO	Gap
C_60_F_2_	–	0	–	–	3.95	−5.86	−3.34	2.52
C_60_F_2_ + drug	0.41	0.01	0.04	0.46	5.61	−5.54	−3.28	2.26
C_60_F_48_	–	0	–	–	0.17	−9.51	−4.15	5.36
C_60_F_48_ + drug	0.29	0.03	0.06	0.38	7.01	−5.37	−4.16	1.21

**Table 2:** Calculated geometric and energetic characteristics of metal-decorated pristine and fluorinated fullerenes. Binding energy *E*_b_ (eV), fullerene deformation energy *E*_def_ (eV), mean length of carbon-ion bonds *l*_C–M_ (Å) and the number of such bonds *n* (dimensionless), mean length of fluorine-ion bonds *l*_M–F_ (Å) and the number of such bonds *m* (dimensionless), dipole moments *D* (Debye), frontier molecular orbitals HOMO and LUMO (eV) and HOMO–LUMO gaps (eV) are presented.

System	*E*_b_	*E*_def_	*l*_C–M_ (*n*)	*l*_M–F_ (*m*)	*D*	HOMO	LUMO	Gap
non-fluorinated fullerene								
C_60_Cr^3+^	17.94	0.18	2.265 (3)	–	18.79	−7.08	−6.14	0.94
C_60_Fe^2+^	6.26	0.39	2.013 (2)	–	14.85	−6.85	−5.83	1.02
C_60_Fe^3+^	21.71	0.35	2.034 (2)	–	15.17	−7.14	−6.20	0.94
C_60_Ni^2+^	8.69	0.44	1.928 (2)	–	9.45	−6.91	−6.00	0.91
low-fluorinated fullerene								
C_60_F_2_Cr^3+^	21.97	–	2.150 (1)	1.684 (2)	1.87	−7.13	−6.90	0.23
C_60_F_2_Fe^2+^	8.54	–	2.046 (1)	1.836 (2)	2.84	−5.96	−5.03	0.93
C_60_F_2_Fe^3+^	22.67	1.16	1.994 (1)	1.866 (1)	12.18	−7.14	−6.29	0.85
C_60_F_2_Ni^2+^	9.04	0.89	1.924 (1)	1.895 (1)	10.98	−6.93	−5.79	1.14
high-fluorinated fullerene								
C_60_F_48_Cr^3+^	14.02	2.30	–	2.023 (3)	29.81	−9.37	−7.94	1.43
C_60_F_48_Fe^2+^	4.64	1.06	–	1.918 (3)	39.71	−9.92	−8.30	1.62
C_60_F_48_Fe^3+^	17.01	2.62	–	1.921 (3)	26.77	−9.40	−8.47	0.93
C_60_F_48_Ni^2+^	3.89	0.55	–	1.930 (2)	39.48	−9.92	−9.56	0.36

**Table 3:** Calculated geometric and energetic characteristics of metal-decorated pristine and fluorinated C_60_ loaded by chloroquine drug. Binding energy *E*_b_ (eV), fullerene and drug deformation energies *E*_def_ (eV), bond lengths between the metal ion and nitrogen atom *l*_M-N_ (Å), dipole moments *D* (Debye), frontier molecular orbitals HOMO and LUMO (eV) and HOMO–LUMO gaps (eV) are presented.

	*E*_b_	*E*_def_ (Fullerene)	*E*_def_ (Drug)	*l*_M-N_	*D*	HOMO	LUMO	Gap
non-fluorinated fullerene								
C_60_Cr^3+^ + drug	5.21	0.16	0.79	1.968	39.53	−7.04	−4.42	2.62
C_60_Fe^2+^ + drug	3.74	0.25	0.68	1.904	20.39	−5.18	−4.55	0.63
C_60_Fe^3+^ + drug	5.20	0.23	0.84	1.909	39.81	−6.58	−4.67	1.91
C_60_Ni^2+^ + drug	2.61	0.23	0.32	1.853	28.88	−5.66	−5.33	0.34
low-fluorinated fullerene								
C_60_F_2_Fe^3+^ + drug	4.85	0.32	0.43	1.908	34.46	−7.01	−5.24	1.77
C_60_F_2_Ni^2+^ + drug	3.43	0.04	0.24	1.852	15.30	−6.03	−5.07	0.96
high-fluorinated fullerene								
C_60_F_48_Cr^3+^ + drug	8.01	0.87	0.65	1.895	68.71	−6.41	−6.27	0.14
C_60_F_48_Fe^2+^ + drug	4.78	0.87	0.58	1.875	44.13	−5.38	−4.97	0.41
C_60_F_48_Fe^3+^ + drug	9.32	1.06	0.80	1.868	68.96	−7.10	−5.32	1.78
C_60_F_48_Ni^2+^ + drug	5.88	0.47	0.42	1.846	46.82	−5.90	−5.46	0.45

## Data Availability

The data is available as Supplementary Materials to this article.

## Supplementary Materials

The following supporting information can be downloaded at: https://www.mdpi.com/article/10.3390/ijms23042345/s1. Reference [54] is cited in the supplementary materials.
