# Supplementary material for: Ab Initio Insight into the Interaction of Metal-Decorated Fluorinated Carbon Fullerenes with Anti-COVID Drugs
Source: Int J Mol Sci. 2022 Feb 21;23(4):2345. doi: 10.3390/ijms23042345 (PMC8879019; doi:10.3390/ijms23042345)
Supplement: Supplementary file 1 [file ijms-23-02345-s001.zip › ijms-1585476-supplementary.pdf]

Ab initio insight into the interaction of metal-decorated fluorinated carbon fullerenes with anti-COVID drugs

Konstantin P. Katin[1,2,*], Alexey I. Kochaev[1,3], Savas Kaya[4], Fadoua El-hajjaji[5], Mikhail M. Maslov[1,2]

[1] Laboratory of Computational Design of Nanostructures, Nanodevices, and Nanotechnologies, Research Institute for the Development of Scientific and Educational Potential of Youth, Aviatorov str. 14/55, Moscow 119620, Russia;
[2] National Research Nuclear University "MEPhI", Kashirskoe Shosse 31, Moscow 115409, Russia;
[3] Research and Education Center "Silicon and Carbon Nanotechnologies", Ulyanovsk State University, 42 Leo Tolstoy Str., Ulyanovsk 432017, Russia;
[4] Department of Chemistry, Faculty of Science, Cumhuriyet University, Sivas 58140, Turkey;
[5] Engineering Laboratory of Organometallic, Molecular Materials, and Environment, Faculty of Sciences, University Sidi Mohamed Ben Abdellah, Fez 1796, Morocco.

* Correspondence: KPKatin@yandex.ru (Konstantin P. Katin)

SUPPLEMRNTARY MATERIALS

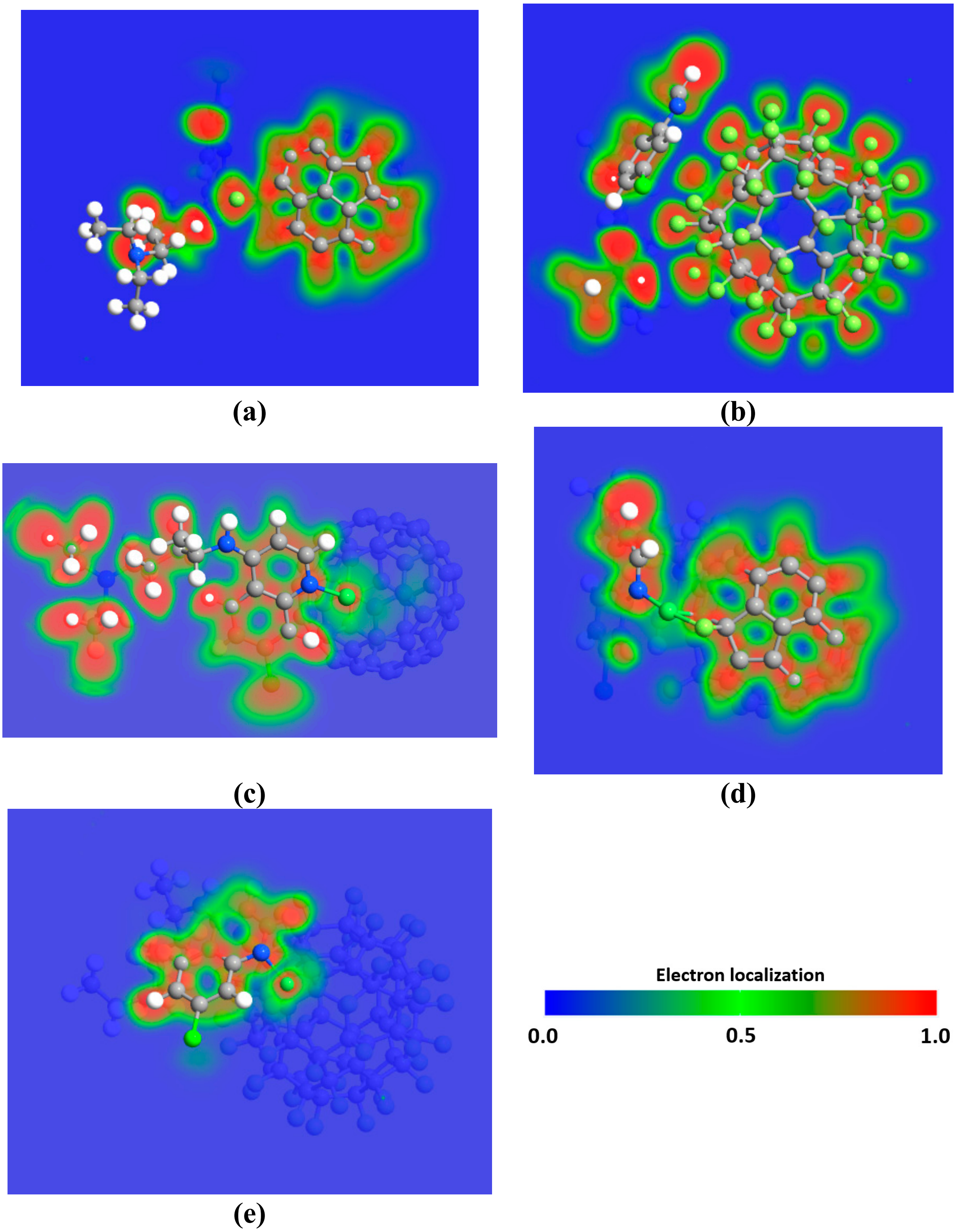

Figure S1. Electron localization function calculated for chloroquine drug loaded on (**a**) $C_{60}F_2$, (**b**) $C_{60}F_{48}$, (**c**) $C_{60}Ni^{2+}$, (**d**) $C_{60}F_2Ni^{2+}$, and (**e**) $C_{60}F_{48}Ni^{2+}$. Figure is plotted with Quantum ATK software [54].

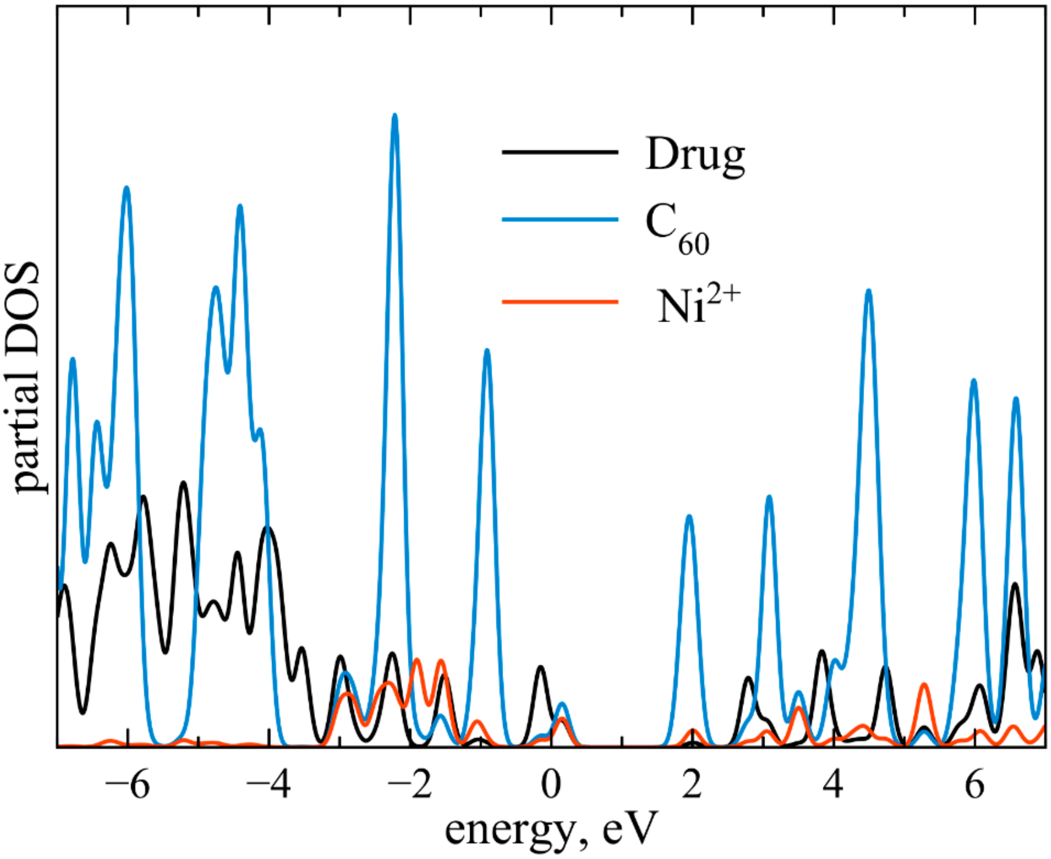

Drug
C_{60}
Ni^{2+}
partial DOS
−6
−4
−2
0
2
4
6
energy, eV

(a)

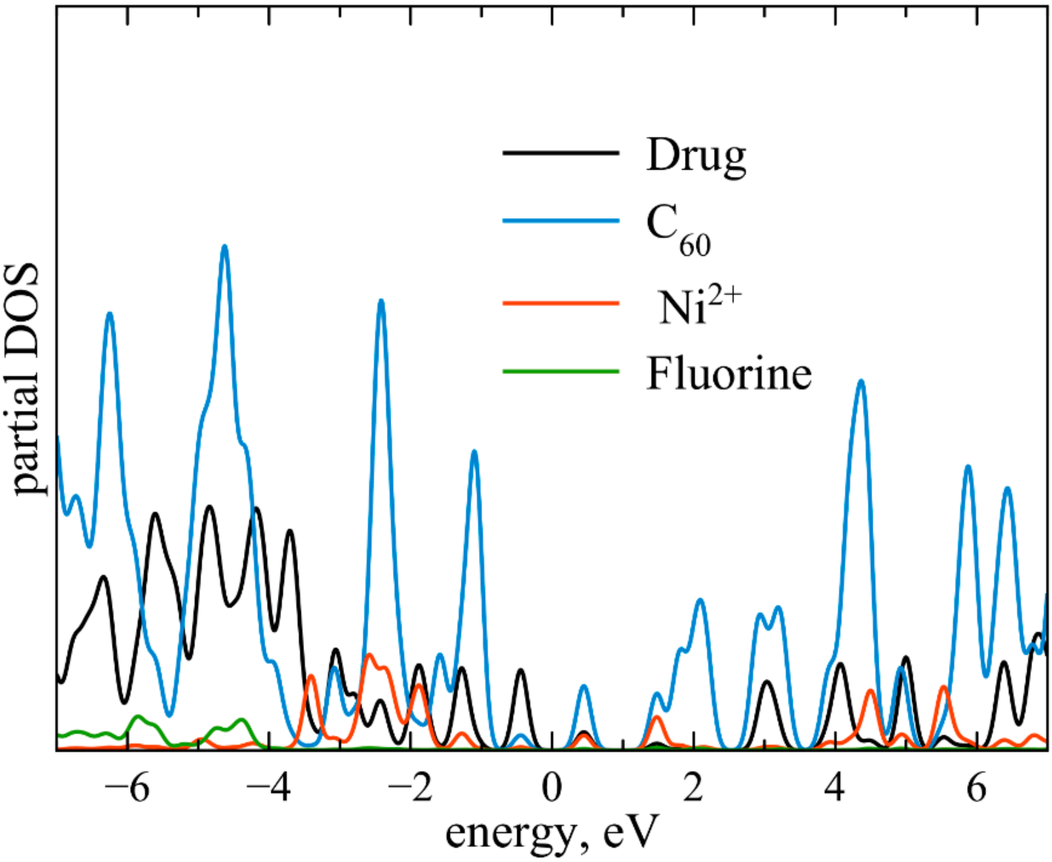

Drug
C_{60}
Ni^{2+}
Fluorine
partial DOS
−6
−4
−2
0
2
4
6
energy, eV

(b)

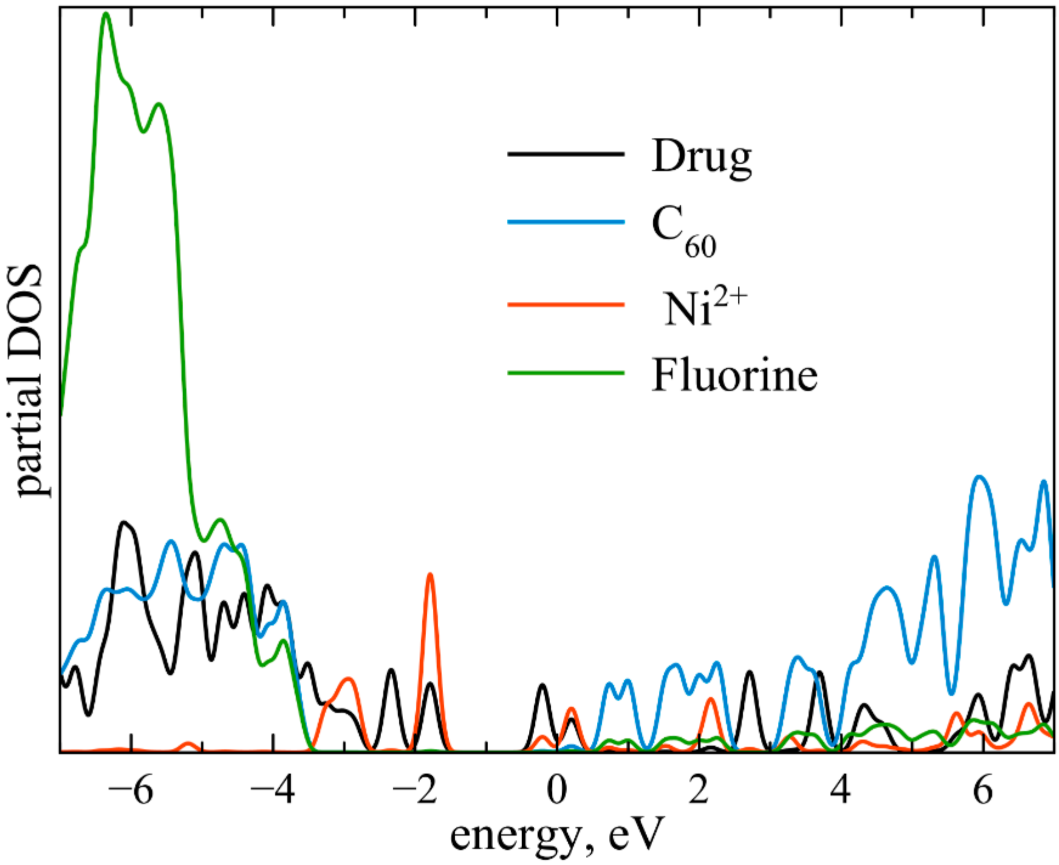

(c)

Figure S2. Partial densities of state calculated for chloroquine drug loaded on (**a**) $C_{60}Ni^{2+}$, (**b**) $C_{60}F_2Ni^{2+}$, and (**c**) $C_{60}F_{48}Ni^{2+}$. Zero energies correspond to the arithmetic mean of the frontier HOMO and LUMO orbitals. The calculated energies of molecular orbitals are broadened by Gaussian curves with $\sigma = 0.1$ eV.
